# Effective Educational Strategies to Promote Life-Long Musical Investment: Perceptions of Educators

**DOI:** 10.3389/fpsyg.2018.01977

**Published:** 2018-10-25

**Authors:** Amanda E. Krause, Jane W. Davidson

**Affiliations:** ^1^The Melbourne Conservatorium of Music, The University of Melbourne, Parkville, VIC, Australia; ^2^School of Psychology and Speech Pathology, Curtin University, Perth, WA, Australia

**Keywords:** music education, musical investment, life-long, music participation, self-determination theory

## Abstract

While research has broadly considered the wide-ranging intellectual, social, personal, and physical benefits of active musical participation across the lifespan, there is little research that explores how music educators work to promote participant investment inside school and beyond. The present research, therefore, aimed to investigate the practices employed by leading music educators within a range of cultural and pedagogical contexts that facilitate investment toward life-long engagement in music. Interviews with North American, European, and Australian music educators with both practitioner and research expertise from within school as well as higher education institutions were undertaken to gather reflections on participants’ own practices and beliefs. Content analysis of the interview transcripts revealed deep knowledge and skills relating to teaching music, education philosophy and pedagogy, and strong recognition of the support of peers, supervisors, institution/school, and local community. It was clear that interviewees were deeply influenced by local, national, and cultural trends. Further, the advice they offered for new/beginning music educators was to think beyond the structure of their own music education and to explore culturally diverse educational experiences for students. Educational approaches that fostered co-production were favored, thus guiding students in their pursuits in learner-directed environments. While the beliefs and practices described are not “new” – echoing well-established educational philosophies – all interviewees argue for a shift from the prevailing pedagogical practice based on expertise training to the promotion cultural connectedness and sharing in and through musical experience. These findings are discussed in terms of Self-Determination Theory, to provide a framework for how music educators can facilitate long-term musical investment through the development of autonomous engagement to generate personal meaning and value in music, which can translate to deeper, longer musical investment. Exploring these pedagogical practices and beliefs in terms of Self-Determination Theory is a significant addition to the literature, enabling the consideration of the type of motivation required to stimulate and develop long-term interest in music.

## Introduction

As McCrary (2001, p.29) remarked, “music educators and researchers should be tireless in their efforts when seeking answers to vitally important questions [including] are we providing outstanding music experiences that foster life-long participation in music”? Research evidence supports the benefits of musical participation across the lifespan. This includes intellectual transfer effects in earlier life (Henriksson-Macaulay and Welch, 2015; Williams et al., 2015) as well as broader benefits to socio-emotional and physical health and well-being into old age (Krause et al., 2018). Accordingly, the present research asks: What are the music educators’ practices for promoting on-going musical engagement? Are there practices appropriate to different forms of music education that serve different cultural contexts and life stages?

Some published work has offered advice and/or strategies regarding teaching strategies to promote student competency and success in general (e.g., Cooper, 2015) as well as strategies pertaining to specific components to music education, such as improvisation (e.g., Azzara, 1999) and practice (e.g., Barry and McArthur, 1994). Cooper’s (2015, p. 3) suggestions, for instance, include providing students with foundational knowledge, opportunities for performance, collaboration, and reflection, and encouraging both creativity and critical thinking. This work is very limited, however, as it does not address promoting students’ on-going musical engagement. Another line of enquiry has considered music teachers’ beliefs about and experiences of professional development (e.g., Bauer, 2007; Conway and Holcomb, 2008), preservice/first-year music teachers’ concerns and needs (e.g., Fredrickson and Hackworth, 2005; Hourigan and Scheib, 2009; Berg and Miksza, 2010; Miksza and Berg, 2013), and identity development as a teacher (e.g., Dolloff, 1999; Haston and Russell, 2012). This work, including the investigations regarding the types of skills and characteristics of successful and effective teaching particularly (e.g., Taebel, 1980; Teachout, 1997; Mills and Smith, 2003; Rohwer and Henry, 2004; Davis, 2006; Miksza et al., 2010), have implications for promoting students’ on-going musical engagement, even with their emphasis on teacher advancement, training, and retention rather than student engagement. Enthusiasm and energy and good classroom management appear to be critical to successful teaching for instance – as they were the two highest ranked characteristics and skills in Miksza et al.’s (2010) study. Interestingly, the findings from multiple studies (Teachout, 1997; Mills and Smith, 2003; Davis, 2006; Miksza et al., 2010) indicate that personal and teaching skills and characteristics are perceived as more important than music skills. However, identifying particular strategies and skills that will specifically promote students’ continued investment remains absent in this previous work.

One area of significant research concerns teacher success by focusing on musical achievements of younger school-aged students (Klinedinst, 1991). This work is limited since it is achievement outcome focused, for example, a score on an aptitude test, a certain score at a competition, or a particular performance. Furthermore, much of the research on continued involvement in music is specifically focused on recruitment into classroom music education (e.g., Abeles, 2004; Albert, 2006), student retention – and drop out – in school music programs (e.g., Corenblum and Marshall, 1998; Adderley et al., 2003; Baker, 2009; Brown, 2012), and retention at school transition points (e.g., Ayling and Johnston, 2005; Bruenger, 2009). Some research has considered the difference between students who persist or drop out of instrumental lessons (e.g., Pitts et al., 2000; Costa-Giomi et al., 2005), but again its focus has been on school-aged participation.

Research considering the role of educators has also considered the students’ musical development, with no attention being given to the teachers’ perspective. The measured outcome – musical development – is framed with regard to success as defined by instrumental mastery, or musical ability (e.g., Davidson et al., 1996, 1998; McPherson and Davidson, 2002; Sichivitsa, 2007; McPherson, 2009). While this research does highlight the influence of educators and key family members in a student’s development, it does not examine the teacher’s motivations or approaches.

The idea of continuing musical involvement seems of particular relevance to education when it is known that musical engagement can result in benefits to other curriculum performance (Costa-Giomi, 2014) and can also lead to an investment in music to offer routes to socio-emotional engagement and well-being across the lifespan. Indeed, a goal of music education has been to inspire students to continue participating in music into the future (Sichivitsa, 2002; Cavitt, 2005; Mantie and Tucker, 2008; Silveira, 2013; Bowles et al., 2014). However, as Green and Hale (2011, p. 46) stated, “continued participation [across the lifespan] is realized less often than music educators might wish” and they urged researchers to consider what might influence continued participation. Framed in terms of Achievement Goal Theory, they offered suggestions to promote a learning orientation amongst students (in order to foster motivation) that include: using varied, meaningful, and challenging tasks; emphasizing effort, improvement, and enjoyment rather than competition during evaluation tasks; and providing opportunities for student input and choice (Green and Hale, 2011, p. 47). The work considers the learning environment and music-making ‘culture’, which as Paparo (2013) discussed, influence whether and to what degree students are involved in music-making – both in and out of school. Paparo (2013, p. 35) argued that educators should incorporate practices that “enable students to be autonomous at each level of their development” to assist in promoting life-long musical involvement. It seems that educators should encourage their students to make music beyond the classroom – and to focus on aspects of the classroom program that will promote continued participation as well as promote links to the learning outside of school (Silveira, 2013).

A disconnect exists, then, between an acknowledgment that music education should be focused on promoting life-long engagement in music and the existing research that has concentrated on early success, school music achievement, and then continued involvement in music as a separate field of enquiry. The present study is designed to explore **how** music educators in a range of contexts can support investment **both inside school and beyond**, across the lifespan. The research specifically asked, what practices facilitate investment toward life-long engagement in music from amateur to a full professional performance career? This question was investigated within a range of cultural and pedagogical contexts that shape the music educator’s work, thus “music educator” was definitely broadly to include those working in school settings, community contexts, and in therapeutically focused work.

## Materials and Methods

### Sample

The study was based on interviews with leading North American, European, and Australian music education experts, selected through purposive sampling, all with at least 20 years of experience. This was set as a benchmark to ensure long practice and engagement with theory. In order to consider the range of music and pedagogical context that shape music educator’s work, we included higher education theorists and practitioners from school, music therapy, community musicians, and performance backgrounds. In particular, the sample includes 12 participants, including a higher education level music educator with 35 years of experience, a former higher education music educator, back in the classroom, running a very large music department in a school from K-Year 12, with 25 years of experience, two music teachers working in secondary education, a higher education level community music educator, two community music facilitators, a higher education music therapist, two practicing music therapists, a higher education performance educator, and two performers.

### Procedure

Informal open-ended interviews (Bhattacharya, 2017) were conducted with the participants. This type of interview is conducted in a conversational style and is based on exploring key ideas without a set of structured questions (Bhattacharya, 2017) – adapting the conversation to the experiences of each interviewee. Each interview began by asking the main research question, but then was tailored to each participant’s experience to allow for deeper exploration. The primary interview questions focused on the interviewee’s experiences, motivations, examples of practice and grounding for these. As such, the data collected was based on the participants’ reflections of their own practice and beliefs.

### Data Analysis

Working within a grounded theory framework, interview transcriptions were analyzed for emergent areas of interest (in line with Bryant and Charmaz, 2007; Saldaña, 2015). In line with grounded theory, the exploration began with an analysis of the interview data, and then, working from these data, appropriate theoretical models were explored.

## Results

Interviewees were all highly informed of research that defined music’s potential and all had strong beliefs regarding its usefulness to life. Perhaps, the most clarifying statement came from the music education professor:

The arguments have always been...like in nineteenth-century England, that you did music for moral cohesion, that your...young people could actually learn to sing hymns, and appreciate that kind of religious aspect of life that was seen as somehow cementing the workforce, if nothing else...But I think more and more, the evidence is...that this musicality is by human design and so means that we have a moral responsibility to invest in music, because it’s part of what it means to be human. And therefore, if it’s part of what it means to be human, then people will do it anyway, which is fine. But from an education system point of view, should you nurture it? And so, the argument is yes you should because it’s part of human design...and therefore, if it is going to be part of the design, are there any other reasons for doing it? And the current debate surrounds, you know...“music for music’s sake” as opposed to something else’s sake...

This discussion stresses how modern Western society has regarded music and how its educators approach music participation – its benefits being in and of itself, and also for other positive outcomes such as improving well-being and social cohesion. While philosophical debate surrounding the benefit of and/or reason for a music education has a history that stretches back to the nineteenth century and earlier, it is also clear from the interviewee, that music educators are still grappling with why and how music should be engaged with in school and broader contexts.

### Best Practice Skills

Our analysis revealed five major thematic area relating to how music education can best facilitate long-term music investment (see Table 1). These were expressed in terms of the skills music educators possessed and used in order to promote investment and engagement in music. Each of these is discussed in turn below.

**Table 1:** Music educator skills.


Understanding and using a range of musics
Other peoples’ music/finding authentic voices
Strong, sensitive and attuned to provisional knowledge
Letting the learning happen: activating opportunity
Engendering commitment


#### Understanding and Using a Range of Musics^1^

One of the professors training music teachers commented:

Research data tells us that 90% of the people that are graduate musicians will earn a living outside of Western classical music. So, they’ll be doing lots of other things, either in the music industry or not. If they’re performing, it’s often other kinds of music. So in one sense, the Western classical model provides them with a grounding, a set of skills, but in another sense, looking across musical genres, you discover that you come out of the Western classical tradition also with lacks...**a lack of knowledge of how to just make music...make music for fun...be creative, to be able to improvise, to be able to write music, to make music in the moment with other people**. You can’t do it. You find it incredibly difficult. It colors the whole system.

This view suggests that educators and facilitators need to have experienced music as performers, creators, *and* listeners. Also, there is a need to understand how music works beyond the Western classical tradition. Educators need to be equipped with a set of skills that the Western classical training does not offer. Training needs to involve broadening their musical scope and thinking.

A senior music therapist working in higher education noted:

Musical thinking is strategic...in terms of the architecture of “where I need to go next”...to address the needs of this client...it needs strategic music therapist thinking, which is asymmetrical...The music therapist is taking the professional responsibility for strategic direction of where the music is going...That is, part of quite long-term training, in terms of what those strategies might be and what they’d be for, why you are doing it, when are you doing it, when’s your timing..., and what’s the quality of your listening, such that you’re just looking for an opportunity to do something...

So, these skills have to be learned. This includes drawing on appropriate tools for the area of concern (e.g., therapy versus classroom music).

Repertoire was raised as one vital area for motivating participants, as a community musician commented:

I would offer them a selection [of repertoire] that they could choose from. I don’t simply say “What do want to sing?” because I thought that would be too hard to then prepare it. But if they want to sing a whole lot of stuff...I wanted them to have some benefits, and it has to be engaging for me too...so if they just wanted to sing stuff that I had no connection with, game over. I couldn’t stand it. So, I choose a selection and say “What do you want to sing within this?” So...it wasn’t collegiate, in that I asked them to choose with me...it was consultative, a little bit.

Thus, the interviews provide evidence that teachers, facilitators/trainers believe that people in receipt of musical education or therapeutic experience need to be challenged a little, whether child or an older person, always in a culturally/context specific manner.

#### Other Peoples’ Music/Finding Authentic Voices

One of the facilitators explained that she had to research repertoire appropriate to her Jewish clients, offering a mix of old familiar material, but also offer new works to challenge them (and stimulate her). The interview data revealed that across the entire sample, the music educators consistently aimed to encourage awareness and sensitivity to other styles and find a connective voice to express this knowledge.

The Music Therapy lecturer commented:

**I need to keep to my musical voice while being mindful of other traditions**...

It requires a...level of communicative musicality...

...you have to be **authentically in your own music**...

...what are the possibilities that our personal worlds can have **an authentic meeting**..

But this was far more subtle knowledge that having some stock Eastern European Folk melodies or some African drumming patterns in a “bag of tricks,” as a community music lecturer stated:

The musicians must be **authentic**: true to themselves and able to **open their ears and their hearts and be as clear as possible in their musical, emotional, verbal, and non-verbal exchanges.** Some people find a way to opening their musical voice **through exercises**, using a wide range of musics from around the world, improvising and so on. The externalization of self often need skills training to: the **performativity of being a community musician**. Being open to accept people and allowing them full access to your facilitator potential. **It is about tolerance, compassion, but also not being downtrodden or exploited.** It is a leadership role, so you also need to be confident.

It is clear that if we are looking for best practice, superficially dabbling in range of musics is not helpful. Rather, care needs be taken to contextualize and support the incorporation of various musics in an appropriate and knowledge-grounded manner.

#### Strong, Sensitive and Attuned to Provisional Knowledge

The Head of Music in a secondary school and former university educator commented on the delivery of music education training:

[We] try to find schools to put students in schools that have teachers that will demonstrate that kind of **sensitivity**, that **empathy**, **differentiation**...**to take whatever knowledge they’ve got, and take it forward**, and at the same time, we will use things like video, research, published papers, to get them [the students] to think about two things which are very closely related, but which aren’t identical: **research-based teaching, research based evidence, but also the craft of it, to make sure that these two things are constantly being challenged.** The things that are apparent certainties...when you talk to the experts, they aren’t. They’re fuzzy. And therefore, **we want the rounded education which enables people to engage with knowledge, and advanced knowledge, in a way that recognizes that knowledge is provisional**, you know, in the way that the expert does.

This opinion seems based on developing a craft of teaching. It is clear that this type of endeavor is demanding. As the trainer of music therapists noted:

[We need to train music therapists to] the fine tuning of the musical work...the key to that lack of fine-tuning...is related more to their listening than their playing...We often need to get them to stop doing what they’re used to do in terms of providing music for people with a lot of structure...and learn to listen in very great detail to the potential of what that person could do if there’s more space...that waiting...listening...is very hard to learn.

Thus, practice requires delicate monitoring and development. For music therapists this entails:

...**reflexivity**. That’s based on, right from the beginning, taping sessions-let’s say audio taped- and listening back to what you did do in the session in real time and having a kind of protocol for listening and reflecting.

Traditionally, this is quite difference to the skills of performing the instrument well, it is about coordinating, meeting, and moving the people. But, as a music teacher noted:

One of the best ways to improve communication and indeed technique, is for the student to see and hear themselves in practice and concerts. That kind of reflection helps a lot.

Training music educators and community musicians is certainly similar, for they too need to attune:

It’s all about find a way to make a musical contact with a person and then developing that musical contact into some kind of shared structure, and shared musical understanding...It requires time, and the development of musical confidence.

This might be conforming to a prescribed set of principles or a curriculum, or it could be much freer, as the next theme highlights.

#### Letting the Learning Happen: Activating Opportunity

Music educators can either be straight-jacketed by the curriculum, or be subversive as these three expert and highly acclaimed teachers reveal:

Group of three teachers discussing:

A: ...you can **chuck things at them in different ways**... [women composers…saxophone baroque examples]
B: …that’s how we learned about transposition actually...that’s a skill they need to know but this way **they actually had to figure it out so that they could get their ensemble to function**, not because it was on the curriculum to do...
A: I think giving them **good musical problems to solve** is it... [example]
C: ...if they are doing instrumental lessons, to bring that into the classroom...bring your trumpet into the classroom, let’s make it part of it...
A: and that’s a very new thing...suddenly the kids are rocking up to their classroom with their instruments expecting that they might be using it...

As the Mid-career secondary teacher [B] continues:

[As educators we need to learn that] the **best thing we can do is actually to get out of their way**...**point them in a direction they’re wanting**...but don’t put an obstacle for them or try to stop them...or “why don’t you come and join”..

Here, the educators highlight how their students need space to problem-solve, experiment, and grow as musicians and learners. There is recognition of student-centered pedagogy, allowing the students through the provision of an activity to take charge and make progress. Community music facilitation can also have an emphasis on permitting freedom to explore, alongside offering support to develop specific skills. This kind of support may take less of an educational focus, in order to focus on providing strong socio-emotional support and benefits:

Community musicians:

I always believe in **fun**, but I **challenge them a bit too. Not too much though**, as I want them to feel safe. You begin with simple things like breathing, then a bit more technique and them eventually, you can vary the repertoire from very straight forward unison songs, to parts and rounds. It takes a long time to **build their confidence** and **develop the dynamic** in a way that they will rise to the new challenges that I set. You learn to follow a routine to give people a structure and a formula. Sometimes I get complaints if I don’t do things the same as usual.

Oddly enough, I think the overriding skill is **laughter, and humor**. I’m not a wildly funny person, but I seem to have a quirky view on things, and people respond well to that. And I think that **allows us to relax** without me doing what I recall from my singing lessons, having a singing teacher standing over me going “RELAX!,” which is going to do anything but make me relax. Whereas, if you drop the people into a more relaxed zone, via a joke about yourself, or a flippant remark about something that you’ve seen, **they’re in a different headspace, and a better place to take on board some of the other things.** Um...and **there’s that laughter, and there’s also attention to detail**…and normally I’m prepared before I go into a session, and I want them to work on something. I need to ask something of them. You can’t just sing robotically. If I give them a task, and we go over that task repeatedly, and **I endeavor to get something from them**, I think people respond to that so much better than just singing the song through, then “next one!”...But when you actually demand something of them, **it stimulates something in them that makes them feel a sense of achievement**, so...yeah. Those are two big things that I bring to my sessions.

#### Engendering Commitment

The fifth theme that emerged pertained to engendering a commitment to the musical activity. The educator participants demonstrated an awareness of broader goals beyond curriculum or facilitator aims. Indeed, there was general recognition that best educational practices can promote continued musical involvement and investment across the lifespan.

A Head of Music in a school with a university lecturing background noted:

...if the ultimate goal is wanting these people to have enriching musical lives...it’s so incumbent upon us to make sure... that they get the **opportunity to experience the richness of musical experience**, because for me, there’s nothing, you know, in terms of what matters that in terms of our social and personal regulation, you know, what the heck else is there? And is there anything else more important when you look at young people’s lives – the way we use music in that way...You might have some experiences that might enable them to find their niche, the things that really light their candle musically...**to know the options are huge**...you can’t be skillful at all of those things, but **you can be passionate**

...which is why that skillset is quite hard to define...because you may be a master of some of the things that they want to learn but you may actually not be a master at all of some of the things they want to learn..

We’re supposed to be teaching in terms of the curriculum – you know, the knowledge and the skills...We start with where the students are and their passions and their skills...So instead of starting from a knowledge and skills end, you know, starting from a **motivation and engagement** end...it’s not exactly rocket science but seems to me fundamental to what we’re trying to do...and results have been...hugely impressive.

Here, there is recognition that teaching a curriculum may not promote engagement or motivation to continue with music after the class is over. The separation between the requirement to deliver knowledge through a curriculum and also to motivate students is stark: the two elements do not coincide. It may be that the other four skills described are interwoven within this theme. As the participant remarked, this is not “rocket science.” The idea of engaging and developing both educator and student passions and motivations captures the essence of music education and its function.

## Discussion

### Summary of Interview Findings

These findings provide initial evidence of how music educators can better facilitate student long-term musical investment by developing the promotion of autonomous engagement to enable the generation of personal meaning and value in music, which translates to deeper, longer musical investment. To do this, the students (whether they be in school, community, or university settings) need to feel competent in the tasks undertaken and the knowledge they are able to share. Furthermore, they need to have a strong sense of affiliation and social connectedness in their activities. While these beliefs are not “new,” they argue for a practice that necessitates a significant shift away from the prevailing instrumental music pedagogy, which is based on expertise training, to an appreciation of connectedness, autonomy, and competency. As such, these elements together are theorized by Deci and Ryan (2000) in self-determination, which is based around the satisfaction of specific psychological needs that if satisfied, lead to increased motivation, learning, and well-being.

### Theoretical Framework

A foundational element of self-determination theory is that it is based on innate needs of competence, relatedness, and autonomy (Deci and Ryan, 2000). Competence captures the capacity to be effective; relatedness concerns being connected socially, and integrated in that social group; and autonomy is defined as feeling that pursuits are self-endorsed, self-governed, and of free will (Deci and Ryan, 2000). Through interactions occurring in a social environment, meeting these needs leads to personal growth, vitality, and well-being (Ryan and Deci, 2002).

Self-determination theory has been widely applied, stemming from educational psychology, and is supported by a growing body of research (Evans, 2015). Indeed, the theory has recently been applied in work concerning both music education (e.g., Evans, 2015; Evans and Bonneville-Roussy, 2016; Valenzuela et al., 2018) and music therapy (e.g., Lee et al., 2016b). Of particular relevance to the present investigation is that motivation is central element of self-determination theory. Given the present study is interested in how music educators can promote sustained interest and involvement in music, the three basic needs and construct of motivation from self-determination theory provide a useful theoretical framework for contextualizing the results.

### Competency

Competency values operate in any learning situation: individuals need to believe they are able attain proficiency (Deci and Moller, 2005). In the case of music, if it is valued and believed to be for all, it follows that each person will assume they have the capacity to achieve competence. The teachers we have encountered all have a belief in motivating students toward engagement and ownership and so hold a belief in competency. However, given the examples discussed above, a shift in goal orientation for participation may be necessary. Those who have not received encouragement to learn an instrument have been found to regard musical capacity as something that is fixed, with musical potential being regarded as something that cannot be improved incrementally (McPherson and Davidson, 2006). However, it would seem that a learning environment that supports incremental skills acquisition can motivate engagement and thus enhance a sense of competence.

### Relatedness

As social beings, human behavior is dependent on strong forms of connection, social expression, and relatedness. The need for relatedness encourages larger social groups to offer mutual protection and sharing and forms a basis for knowledge transfer (see Evans et al., 2013). According to psychological needs theory, people tend to choose activities that are conducive to success in their social world and reject activities that inhibit it (Deci and Ryan, 2000). Evidently, owing to the group nature of much music-making, relatedness is strongly experienced in musical practices, thus enhancing feelings of connection. Research has demonstrated that successful music learning occurs where there is engagement to feed relatedness with others and the sense of connection being strongly mediated by identification to the group (Davidson et al., 2009). The role of relatedness for those new to musical experiences has shown the high degree to which the activity brings group cohesion and relevance even where the activity is novel and with older people (Lee et al., 2016a). The discussions with music educators suggest that best practice teaching must nurture relatedness.

### Autonomy

Autonomy is important in learning situations because it influences the satisfaction of competence and relatedness (Deci and Ryan, 2000). The more individuals are able to internalize regulation, the more they are intrinsically motivated, and therefore more likely to feel autonomous (Deci and Ryan, 2000). Such autonomy led to jazz trumpeter Louis Armstrong learning with only repeated self-stimulated exposure to music and plenty of opportunity for individual practice (Bergreen, 1997). As seen in the present study – especially with regard to activating opportunity – facilitators can promote autonomy in their students which in turn engenders engagement in musical activity.

The data we have collected from music educators supports the need to satisfy psychological needs as expressed in self-determination theory. It could be argued that a goal of the music educator(s) is to develop educational opportunity to fulfill the three basic psychological needs, critical to self-determination.

## Conclusion

In the present study, music educators from a wide range of music sub-disciplines reflected on their practice to consider how we can promote continued musical investment by students from a range of developmental opportunities and stages. Through grounded theory analysis of their interview data, five crucial skills were identified as being necessary to educating for investment in musical pursuits. These include both understanding and being able to use a range of musics to attract people to engage; facilitating the learner to find a personalized understanding and relevance of music; being sensitive and attuned to provisional knowledge; letting the learning happen through provision of opportunity; and engendering commitment in the learner. It was clear that interviewees were passionate about music and the value of music education. Further, educators needed to think beyond their own musical experience and knowledge in order to incorporate additional musics to provide more culturally diverse experiences for their own students. This seemed to involve creating opportunities in which they could guide students in their pursuits via learner-centered and learner-directed environments. When considering these themes and the ideas inherent to their educational practices, it is clear that they reflect educator actions to advocate for the fulfilment of the three basic psychological needs (autonomy, competency, and relatedness), as outlined by self-determination theory.

While drawing on a small number of in-depth interviews, these findings speak to the need to understand how to develop a successful motivationally rich teaching practice. Future research is well-placed to continue investigation. For example, while we interviewed leading music educators, it would be valuable to conduct case study observations of these skills. Through observation study, we could begin to illuminate the ways teaching skills are executed with different aged learners and in different settings (e.g., school versus therapy versus community music setting). With this deeper understanding gained, future research might also examine the prevalence of these practices within a larger group of educators as well as consider their alignment with curriculum and national/cultural demands. We will offer a final recommendation for future research, and that is to consider these educator practices against students’ beliefs and perceptions. Using the self-determination theory, and examining the basic needs, this type of future research could be beneficial in focusing teaching skills around motivating autonomous student learning and commitment.

## Ethics Statement

This study was carried out in accordance with the recommendations of The Human Ethics Committee of the University of Melbourne (Ethics ID: 1442751.1) with written informed consent from all participants.

## Author Contributions

The funding and conceptual work underpinning this article was generated by JD who also developed the interview schedule and ethics and undertook all the interviews. AK developed the literature review, transcribed the interviews, and sketched the shape of the final article. JD undertook the transcript analysis, which was checked by AK and both then co-wrote the discussion and conclusion.

## Conflict of Interest Statement

The authors declare that the research was conducted in the absence of any commercial or financial relationships that could be construed as a potential conflict of interest.

## Footnotes

1 Note that in all cases the emphasis in these quotes is ours to highlight a point/concept.
